# Doppler Parameters of Hepatic and Renal Hemodynamics in Patients with Liver Cirrhosis

**DOI:** 10.1155/2012/961654

**Published:** 2012-07-03

**Authors:** D. Popov, R. Krasteva, R. Ivanova, L. Mateva, Z. Krastev

**Affiliations:** ^1^Clinic of Gastroenterology, University Hospital “St. Iv. Rilski”, 15 Akad. Ivan Geshov Boulevard, 1431 Sofia, Bulgaria; ^2^Clinic of Nephrology, University Hospital “Alexandrovska”, 1 Georgi Sofiiski Street, 1431 Sofia, Bulgaria; ^3^Laboratory of Clinical Pathology, University Hospital “St. Iv. Rilski”, 15 Akad. Ivan Geshov Boulevard, 1431 Sofia, Bulgaria

## Abstract

*Introduction*. There are limited studies on simultaneous evaluation of liver and renal blood flow using Doppler methods. We evaluated and compared the changes of liver and renal Doppler US parameters in patients with liver cirrhosis according to the degree of liver disease. *Material and Methods*. We assessed a large spectrum of liver and renal Doppler US parameters in 67 patients with liver cirrhosis. *Results*. Significant differences between Child's classes or score, as well as MELD score, were observed in all investigated intrarenal blood flow Doppler US parameters, except renal artery peak systolic velocity, but only in some of the hepatic ones. The deviations of renal Doppler US parameters were also related with the complications of liver cirrhosis, as well as serum urea and creatinine levels. There was relationship between Doppler US parameters of hepatic artery and the corresponding renal artery Doppler US parameters. The changes of Doppler US parameters were age independent. *Conclusion*. Our results show, renal Doppler US parameters correlate with the severity and complications of liver cirrhosis. Because of dynamic deviations of renal Doppler US parameters with advance of liver cirrhosis, we recommend Doppler US of renal artery as a part of follow up of these patients.

## 1. Introduction

Portal hypertension is the most important feature of liver cirrhosis and its complications. In addition, kidney dysfunction, especially renal vasoconstriction, plays important role in the hepatorenal syndrome and patient's prognosis [1]. The detailed investigation of the renal blood flow in real time is a challenge for the investigators. Many authors evaluate the hemodynamics of portal vein and hepatic arterial blood flow, as well as renal arterial blood flow [2–20]. There are limited studies on simultaneous evaluation of liver and renal blood flow using Doppler methods in real time [21]. Because of that, the aim of our study was to evaluate and compare the changes of liver and renal Doppler US parameters in patients with liver cirrhosis according to the degree of liver disease. We also assess the relationship between these parameters and some surrogate markers of renal function.

## 2. Material and Methods

A total of 67 in-patients with liver cirrhosis were investigated from 2008 to 2010 year. The main characteristic of the patients with liver cirrhosis was given on Table 1. All patients were treated with Silymarine or vitamins and in addition 39 patients with esophageal varices took beta-blockers (Propranolol), and 42 patients took diuretics (18-Spironolactone, 2—Furosemide, and 22—Spironolactone plus Furosemide). Hypertension (*n* = 27) was the most frequent concomitant disease, but well controlled with the therapy of beta-blockers and/or diuretics. The other comorbidity included diabetes mellitus (*n* = 13), cholelithiasis (*n* = 12), chronic gastritis (*n* = 9), ischaemic heart disease (*n* = 8), chronic bronchitis (*n* = 6), chronic pancreatitis (*n* = 5), chronic gastroduodenal ulcer (*n* = 6), gastro-reflux esophageal disease (*n* = 5), cerebrovascular disease (*n* = 4), diverticulosis (*n* = 2), and others (*n* = 14).

The study was approved by the Institutional Review Committee Board and informed consent for the trial was obtained from each person. The patients with gastrointestinal bleeding and or endoscopic band ligation or sclerotherapy in the last 8 weeks, surgery, thrombosis portal veins, liver tumors, extrahepatic cholestasis, acute alcoholic hepatitis, hepatorenal syndrome, therapy with vasoactive drugs, excluding beta-blockers and diuretics, significant concomitant disease and cotherapy, which might interfere Doppler ultrasound parameters, and renal function were excluded.

Abdominal ultrasound (US), as well as liver and renal Doppler US, was performed and interpreted by one investigator according to standard protocol. US measurements were done by Aloka SSD 4000, convex probe 2.5–5,0 MHz, cut-off filter 100 Hz, with angle of insonation 50°, using a 2–3,5-5 mm Doppler gate, as large as at least one-third of the vessel diameter. The investigated subjects fasted over night and rest in bed in the supine position for at least 10 minutes before the US measurements. Portal vein (PV), hepatic artery (HA), and intrarenal arteries (RAs), interlobar or segmental branch, were evaluated by Color Doppler US. A rectilinear segment of the common tract of the portal vein (PV), as near as possible to the bifurcation, was estimated. The proper hepatic artery (HA) was investigated near the point where it crosses the main PV. A train of at least three similar, sequential Doppler waveforms was obtained during suspended respiration, sometimes in sustained shallow inspiration. Three Doppler waveform tracings were obtained from each kidney by sampling the RA: right (RRA-RA) and left (LRA-RA) in the superior, middle, and inferior portions of the kidney. The mean values of the parameters, for each kidney, were obtained from the measurement of the waveforms of both, right and left renal area.

We evaluated the following hepatic and intrarenal blood flow Doppler parameters (cm/sec): PV peak systolic velocity (PV-PSV), portal venous time-averaged maximum velocity (PVV = PV-PSV × 0,57), HA maximum peak systolic velocity (HA-PSV), HA minimal end diastolic velocity (HA-EDV), HA mean velocity (HA-MnV = HA-PSV × 0,62), HA resistance index (HA-RI = HA-PSV − HA-EDV/HA-PSV), HA pulsatility index (HA-PI = HA-PSV − HA-EDV/HA-MnV), liver vascular index (LVI = PVV/HA-PI), modified liver vascular index (mLVI = PVV/HA-RI), arterio/portal ratio (A/P = HA-PSV/PV-PSV), RA peak systolic velocity (RA-PSV), RA minimal end diastolic velocity (RA-EDV), RA mean velocity (RA-MnV), RA resistance index (RA-RI = RA-PSV − RA-EDV/RA-PSV), and RA pulsatility index (RA-PI = RA-PSV − RA-EDV/RA-MnV).

The diagnosis of liver cirrhosis was based on standard criteria. Besides the standard hematological and biochemical tests, we also tested urine sodium excretion (mmol/24 h), glomerular filtration rate (GFR, mL/min), and correct GFR (GFR × 1,73/body surface, mL/min). For evaluation of the degree of liver damage we used Child-Pugh classification of liver cirrhosis [22] and MELD [23].

Statistical analysis was performed using ANOVA, Mann-Whitney, and correlation analyses (SPSS v.14). *P* value less than 0.05 was considered as statistically significant.

## 3. Results

### 3.1. Liver Doppler US Parameters

#### 3.1.1. Relationship between Liver Doppler US Parameters and Severity of Liver Cirrhosis

From all evaluated liver Doppler US parameters significant differences among Child's class A, B, and C were observed only for the peak systolic velocity of PV, liver, and modified liver vascular indices (Table 2). We also found significant relationship between Child's score and PV time-averaged maximum velocity, PV peak systolic velocity, and liver vascular index values. On the other hand HA mean velocity, HA maximum peak systolic velocity, and arterio/portal ratio were higher in patients with MELD score more than 20 compared to the patients with MELD score less than 20 (Table 3). There was no correlation between values of liver Doppler US parameters and MELD score.

#### 3.1.2. Relationship between Liver Doppler US Parameters and Complications of Liver Cirrhosis

There were no significant differences between liver Doppler US parameters and the presence of esophageal varices and collaterals and among patients with compensated and decompensated liver cirrhosis, as well as patients with or without ascites, or portal encephalopathy.

### 3.2. Renal Doppler US Parameters

#### 3.2.1. Relationship between Renal Doppler US Parameters and Severity of Liver Cirrhosis

All intrarenal blood flow Doppler parameters except right and left RA peak systolic velocity showed significant differences between Child's class A, B, and C (Table 4). In addition we also found a significant relationship between Child's score and right and left RA minimal end diastolic velocity, right and left RA resistance, and RA pulsatility indices.

According to MELD score a significant decrease of right and left RA mean velocity and minimal end diastolic velocity and an increase of right and left RA resistance and RA pulsatility indices were observed in patients with MELD <20 versus those with MELD >20 (Table 5). We also found correlations between those renal Doppler parameters and MELD score. Only values of right and left RA peak systolic velocity showed no significant change.

#### 3.2.2. Relationship between Renal Doppler US Parameters and Complications of Liver Cirrhosis

**Figure 1:**
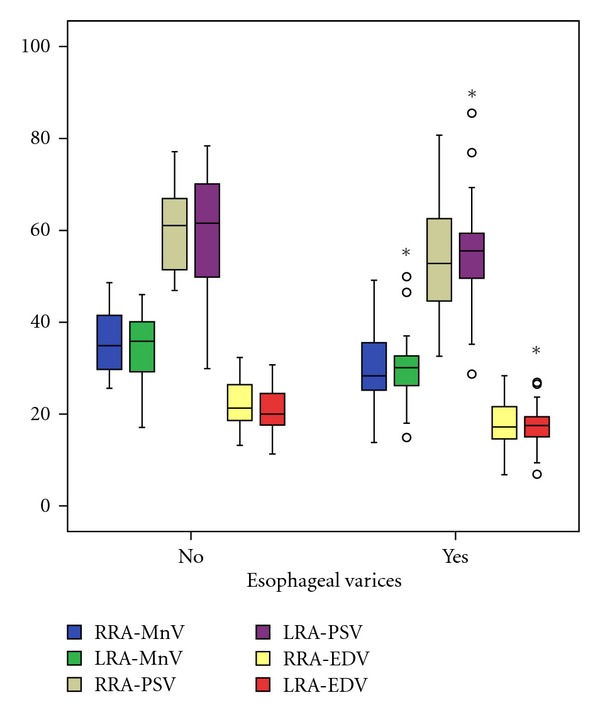
The values of RRA/LRA-MnV, RRA/LRA-PSV and RRA/LRA-EDV in patients with presence or absence of esophageal varices.

 In patients with esophageal varices we observed a decrease of right (*P* = 0.002) and left (*P* = 0.038) RA mean velocity, right RA peak systolic velocity (*P* = 0.045), as well as right (*P* = 0.003) and left RA minimal end diastolic velocity (*P* = 0.025) compared to the values of subjects without esophageal varices (Figure 1). There was no significant difference between renal Doppler US parameters in patients with or without collaterals.

**Figure 2:**
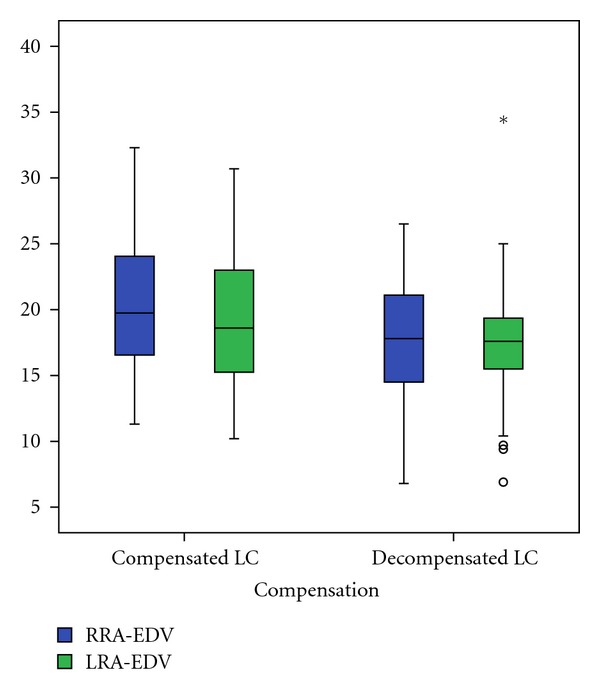
The values of RRA/LRA-EDV in patients with compensated and decompensated liver cirrhosis.

**Figure 3:**
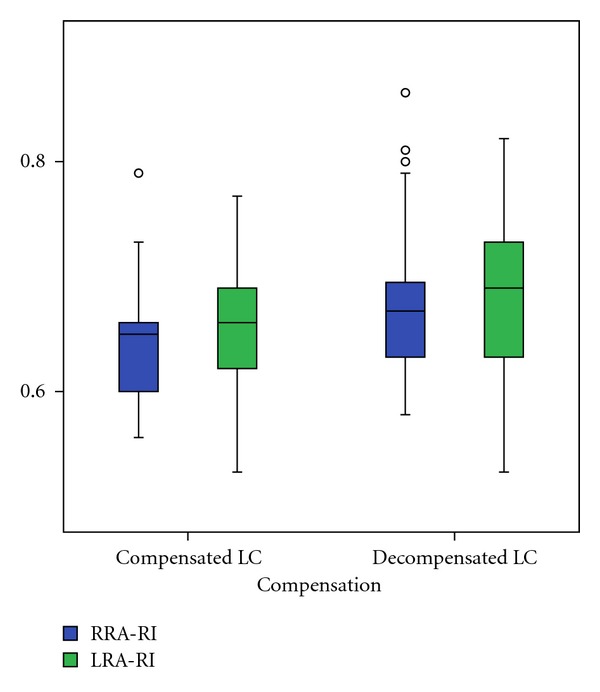
The values of RRA/LRA-RI in patients with compensated and decompensated liver cirrhosis.

**Figure 4:**
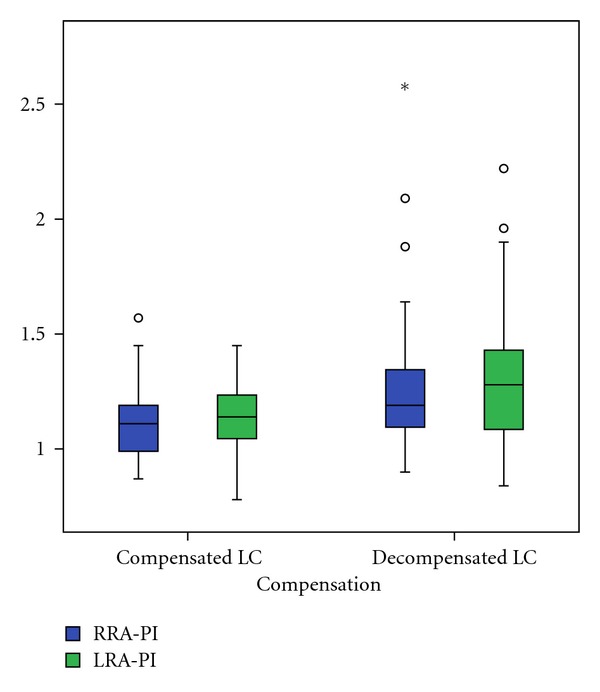
The values of RRA/LRA-PI in patients with compensated and decompensated liver cirrhosis.

**Figure 5:**
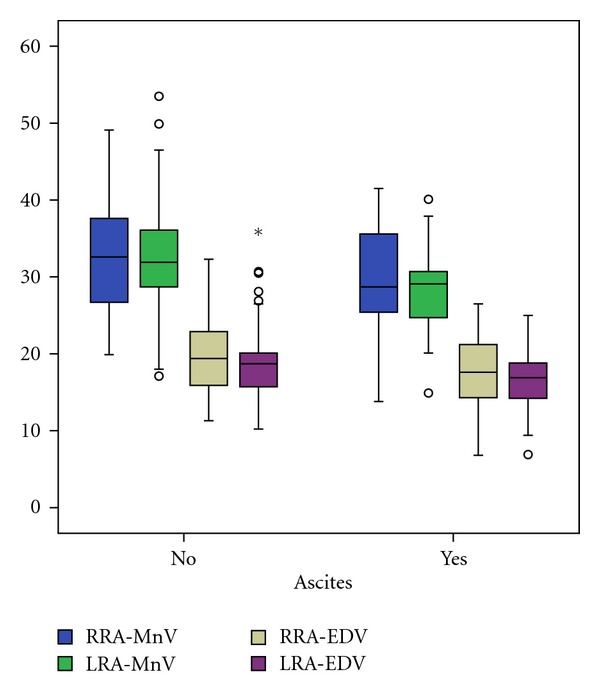
The values of RRA/LRA-MnV and RRA/LRA-EDV in patients with presence or absence of ascites.

**Figure 6:**
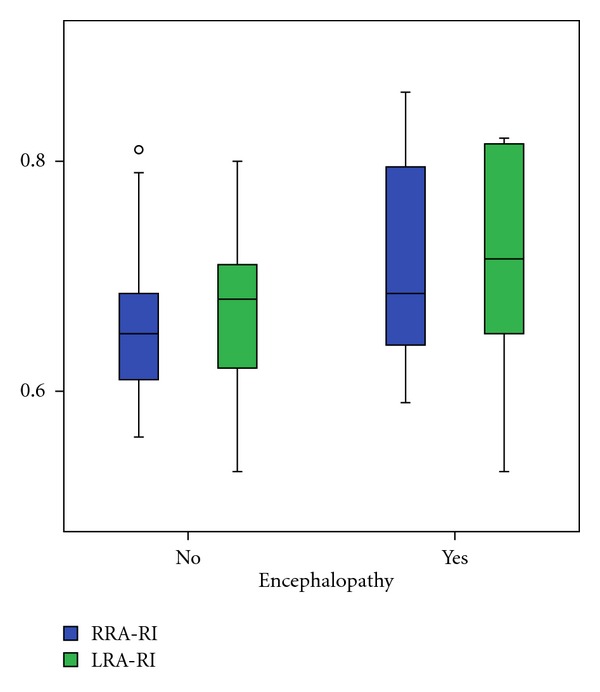
The values of RRA/LRA-RI in patients with presence or absence of hepatic encephalopathy.

**Figure 7:**
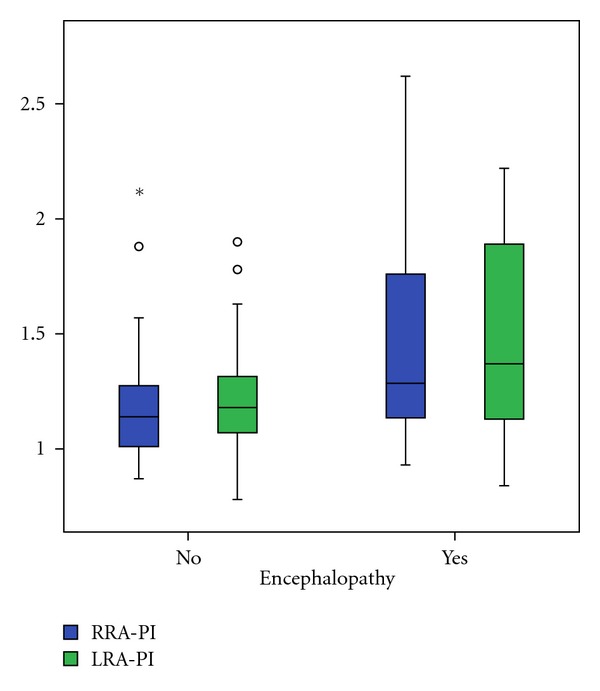
The values of RRA/LRA-PI in patients with presence or absence of hepatic encephalopathy.

In comparison of patients with compensated and decompensated liver cirrhosis we found a significant decrease of right RA minimal end diastolic velocity (*P* = 0.021), as well as an increase of right (*P* = 0.020) and left (*P* = 0.028) RA resistance index and right (*P* = 0.024) and left (*P* = 0.002) RA pulsatility index (Figures 2, 3, and 4). The right and left RA mean velocity (*P* = 0.015) and left RA minimal end diastolic velocity (*P* = 0.027) were also decreased in patients with ascites than those without ascites (Figure 5). In patients with portal encephalopathy we observed significant increase of right RA resistance index (*P* = 0.014), as well as right (*P* = 0.003) and left (*P* = 0.005) RA pulsatility index than subjects without portal encephalopathy (Figures 6 and 7). 

### 3.3. Relationship between Liver and Renal Doppler US Parameters

We observed correlations between Doppler US parameters of hepatic artery and arterio/portal ratio by one hand and some renal Doppler US parameters on the other hand (Table 6).

### 3.4. Surrogate Markers of Renal Function

Serum urea levels and serum sodium levels correlated with Child's and MELD scores (Table 7). Serum urea and serum creatinine levels showed significant correlation with some renal Doppler US parameters, but not with liver Doppler US parameters. GFR, correct GFR, and urine sodium excretion showed no significant relationships.

### 3.5. Impact of Patient's Age on Liver and Renal Doppler US Parameters

We observed relationship between patient's age and some liver Doppler US parameters as HA minimal end diastolic velocity (*r* = −262, *P* = 0.032), liver vascular index (*r* = −240, *P* = 0.05), and modified liver vascular index (*r* = −247, *P* = 0.044). On the other hand, no significant correlation between patient's age and renal Doppler US parameters was found. Among the surrogate markers of kidney function, only the values of GFR (*r* = −571, *P* = 0.0001) and correct GFR (*r* = −536, *P* = 0.0001) showed significant inverse correlation.

## 4. Discussion

Many liver Doppler US parameters and indices have been used to distinguish the patients with portal hypertension from those with normal hemodynamic of portal vein and hepatic arterial blood flow [2–7, 10]. But there are confusing data when comparing their changes according to Child's classes or MELD, as well as the presences of complications of liver cirrhosis [2, 5–7, 9, 24–26]. In this study we evaluated and compared the changes of large number of liver and renal Doppler US parameters, measured simultaneously in patients with liver cirrhosis according to the degree of liver disease. Our results show that PV time-averaged maximum velocity and peak systolic velocity, as well as liver and modified liver vascular indices, are associated with Child-Pugh classes. Some authors also reported the relationship between some liver Doppler US parameters and Child's classes, as well as an appearance of pseudonormalisation of portal vein hemodynamic when liver cirrhosis advances [5–7, 9–12, 24, 25]. However, Child-Pugh score is not the perfect system for the assessment of degree of liver cirrhosis and it has some limitations, including the subjective interpretation of ascites and encephalopathy, and some laboratory deviations, especially prothrombin time. Because of that we also use MELD as one universal system for the assessment of severity of liver cirrhosis [23]. Hepatic artery mean velocity, HA maximum peak systolic velocity, and arterio/portal ratio were higher in patients with MELD score more than 20 compared to the patients with MELD score below 20. On the other hand, no one of the liver Doppler US parameters correlate directly with MELD score. In addition, there were no significant differences between liver Doppler US parameters and various complications of liver cirrhosis. As a whole, our results confirm the buffer role of hepatic artery with advance of liver cirrhosis and pseudonormalisation of some liver Doppler US parameters. One possible reason for the contradicting data in the literature is the heterogenity of patients with liver cirrhosis, included in various studies. The differences also depend on the type of hemodynamic disturbances, for example, the presence of hyperkinetic syndrome or collaterals [5, 9]. Many other factors also interfere with values of liver Doppler US parameters in patients with liver cirrhosis [2, 4, 5, 21]. Acute alcoholic hepatitis and other significant concomitant diseases are exclusion criteria in our study. Despite of that, more than one-third of our patients have an alcoholic etiology of liver cirrhosis, ten patients have collaterals, and more than 50% of the patients take beta-blockers and diuretics. Our data show relationship between patient's age and HA minimal end diastolic velocity, liver vascular index, and modified liver vascular index and confirm the role of age for the deviations of these Doppler US parameters.

The evaluation of the renal hemodynamics in patients with liver cirrhosis is based mainly on the index of resistance of the intrarenal arteries [11–20]. Renal vasoconstriction has been documented in several series of cirrhotic patients on the base of increased resistive index [1]. In patients with refractory ascites, as well as in subjects with serum creatinine within the normal range, increased RI seems to be correlated with a higher risk of subsequent deterioration in renal function [1, 12, 15, 17–20]. In our study in contrast to liver Doppler US parameters, all intrarenal blood flow Doppler parameters except RA peak systolic velocity show a significant association with the severity of liver cirrhosis, evaluated by both Child's and MELD scores. Most of these parameters also correlate with the presence of esophageal varices and ascites, as well as with the decompensation of liver cirrhosis. In addition some renal Doppler US parameters, but no one of liver Doppler US parameters, correlate with serum urea and serum creatinine levels. Similar results were reported when comparing patients with liver cirrhosis with and without ascites and with first signs of renal failure [13, 20]. The data about correlation between RI and the glomerular filtration are controversial [1, 13]. The increase of creatinine level corresponds to the higher values of the impedance indices. Renal artery Doppler US parameters may be useful for identifying patients at high risk for developing impaired renal function at an early stage. On the other hand, there is no evidence that Doppler US helps differentiate cirrhotic patients with impaired renal function only related to vasoconstriction from patients who have both vasoconstriction and intrinsic kidney damage. The values of renal Doppler US parameters also depend on many factors [1, 14]. The application of diuretics leads to an elevation in the impedance indices of the intrarenal arteries. Paracentesis and albumin infusion are followed by a significant decrease in renal RI. Because of that, renal artery Doppler US may help to clarify the role of therapeutic intervention on renal hemodynamics. In our study the therapy of beta-blockers and diuretics, as well as concomitant cardio-vascular disease or diabetes, may interfere with the renal blood flow. On the other hand, our data show no correlation between patient's age and renal Doppler US parameters. These results are in conflict with the data in subjects without liver cirrhosis.

The simultaneous measurement of liver and renal Doppler US parameters allows to find relationships between Doppler US parameters of hepatic artery and the corresponding renal artery Doppler US parameters. These data emphasize common pathophysiological mechanisms for impairment of hepatic and renal artery blood flow. On the other hand, renal Doppler US parameters show more dynamic deviations than liver Doppler US parameters in advance of liver cirrhosis and may need to be evaluated in follow-up of these patients.

In conclusion, our results show parallel changes in renal and hepatic artery Doppler US parameters in patients with liver cirrhosis, independently of patient's age. There is no direct correlation between the degree or complications of liver cirrhosis and most of liver Doppler US parameters. In contrast, renal Doppler US parameters correlate with the severity and complications of liver cirrhosis. Because of dynamic deviations of renal Doppler US parameters in advance of liver cirrhosis, we recommend Doppler US of renal artery as a part of the followup of these patients.

## Figures and Tables

**Table 1:** Characteristics of patients with liver cirrhosis.

Parameters	All patients (*n* = 67)	Child A (*n* = 33)	Child B (*n* = 22)	Child C (*n* = 12)
Age (*x* ± SD)	54.22 ± 12.36	56.85 ± 12.26	51.45 ± 12.75	52.08 ± 11.34
Sex—m/f	39/28	17/16	14/8	8/4
Etiology (*n*)				
Alcohol	25	5	10	10
Viral	37	26	10	1
Study approved	2	1	1	—
Autoimmune	3	1	1	1
Others				
LC (*n*)				
Compensated	32	29	3	0
Decompensated	35	4	19	12
Esophageal varices (*n*)				
Yes/no	49/18	23/10	14/8	12/0
Ascites (*n*)				
Yes/no	26/41	2/31	13/9	11/1
Encephalopathy (*n*)				
Yes/no	8/59	0/33	2/20	6/6
MELD score (*x* ± SD)	15.01 ± 7.17	10.90 ± 3.29	15.32 ± 3.67	25.75 ± 8.64
Therapy with (*n*):				
B-blockers—Yes/no	39/28	15/18	16/6	8/4
Diuretics—Yes/no	42/25	16/17	15/7	11/1
Comorbidity (*n*)				
Yes/no	50/17	28/5	14/8	8/4

**Table 2:** Liver Doppler US parameters in patients with liver cirrhosis according to Child-Pugh classification.

Parameter	All patients (*n* = 67)	Child A (*n* = 33)	Child B (*n* = 22)	Child C (*n* = 12)	Child's score (5–15 points)
	Mean ± SD	Mean ± SD	Mean ± SD	Mean ± SD	Correlation (*r*)
PVV	16.35 ± 3.49	15.68 ± 3.35	16.49 ± 3.03	17.94 ± 4.32	0.268, *P* = 0.028
PV-PSV	28.68 ± 6.12	27.51 ± 5.87	28.93 ± 5.32	31.46 ± 7.59 *P* = 0.045	0.268 *P* = 0.028
HA-PSV	69.60 ± 20.55	70.23 ± 21.07	67.58 ± 16.30	71.56 ± 26.86	
HA-MnV	43.23 ± 12.73	43.49 ± 13.07	42.22 ± 10.09	44.39 ± 16.67	
HA-EDV	23.41 ± 7.13	22.78 ± 7.30	23.86 ± 6.65	24.33 ± 7.96	
HA-RI	0.66 ± 0.07	0.67 ± 0.04	0.64 ± 0.07	0.65 ± 0.10	
HA-PI	1.06 ± 0.12	1.09 ± 0.08	1.03 ± 0.13	1.04 ± 0.17	
A/P	2.50 ± 0.78	2.60 ± 0.73	2.39 ± 0.67	2.43 ± 1.08	
LVI	15.73 ± 4.37	14.55 ± 3.55	16.18 ± 3.36	18.13 ± 6.75 *P* = 0.041	0.282 *P* = 0.021
mLVI	25.21 ± 6.88	25.21 ± 6.88	26.04 ± 5.50	28.47 ± 10.60 *P* = 0.041	

**Table 3:** Liver Doppler US parameters with significant differences according to MELD score.

Parameter	MELD	*N*	Mean	SD	95% confidence interval for mean		Minimum	Maximum	*P *
					Lower bound	Upper bound			
HA-MnV	<20	55	41.32	10.45	38.49	44.14	22.30	71.36	0.007
	>20	12	52.01	18.29	40.39	63.62	24.37	84.60	
HA-PSV	<20	55	66.49	16.85	61.93	71.04	36.00	115.10	0.007
	>20	12	83.85	29.48	65.12	102.58	39.30	136.40	
A/P	<20	55	2.41	0.63	2.24	2.58	1.15	4.07	0.029
	>20	12	2.95	1.20	2.18	3.71	1.01	4.81	

**Table 4:** Renal Doppler US parameters in patients with liver cirrhosis according to Child-Pugh classification.

Parameter	All patients (*n* = 67)	Child A (*n* = 33)	Child B (*n* = 22)	Child C (*n* = 12)	Child's score (5–15 points)
	Mean ± SD	Mean ± SD	Mean ± SD	Mean ± SD	Correlation (*r*)
RRA-PSV	56.06 ± 11.24	57.47 ± 11.07	55.33 ± 11.07	53.53 ± 11.29	
LRA-PSV	55.79 ± 12.36	56.56 ± 13.27	56.69 ± 11.44	52.01 ± 11.62	
RRA-MnV	31.62 ± 7.36	32.73 ± 6.65	32.63 ± 7.96	26.70 ± 6.59 *P* = 0.046	
LRA-MnV	31.10 ± 7.61	31.85 ± 7.79	32.67 ± 7.04	26.17 ± 6.59 *P* = 0.040	
RRA-EDV	19.07 ± 5.40	19.88 ± 4.78	20.07 ± 5.66	15.00 ± 5.01 *P* = 0.013	−0.400 *P* = 0.001
LRA-EDV	18.17 ± 5.33	18.60 ± 5.27	19.45 ± 5.04	14.62 ± 4.80 *P* = 0.031	−0.298 *P* = 0.014
RRA-RI	0.66 ± 0.06	0.65 ± 0.05	0.64 ± 0.05	0.72 ± 0.09 *P* = 0.001	0.456 *P* = 0.0001
LRA-RI	0.67 ± 0.07	0.67 ± 0.05	0.65 ± 0.06	0.71 ± 0.09 *P* = 0.033	0.349 *P* = 0.004
RRA-PI	1.21 ± 0.30	1.17 ± 0.17	1.10 ± 0.17	1.51 ± 0.51 *P* = 0.0001	0.532 *P* = 0.001
LRA-PI	1.23 ± 0.27	1.20 ± 0.21	1.15 ± 0.19	1.47 ± 0.41 *P* = 0.002	0.489 *P* = 0.001

**Table 5:** Renal Doppler US parameters in patients with liver cirrhosis according to MELD score.

Parameter	MELD	*N*	Mean	SD	95% confidence interval for mean		Minimum	Maximum	*P *	MELD score (8–46)
					Lower bound	Upper bound				Correlation (*r*)
	<20	55	32.30	7.13	30.38	34.23	19.90	49.10		
RRA-MnV	>20	12	28.47	7.88	23.46	33.47	13.80	39.90	NS	−0.396 *P* = 0.001
	Total	67	31.62	7.36	29.82	33.41	13.80	49.10		

	<20	55	31.37	7.15	29.43	33.30	17.10	49.90		
LRA-MnV	>20	12	29.89	9.73	23.71	36.08	14.90	53.50	NS	−0.294 *P* = 0.016
	Total	67	31.10	7.61	29.25	32.96	14.90	53.50		

	<20	55	19.80	5.14	18.41	21.19	11.30	32.30		
RRA-EDV	>20	12	15.71	5.46	12.24	19.18	6.80	24.20	0.016	−0.459 *P* = 0.001
	Total	67	19.07	5.40	17.75	20.39	6.80	32.30		

	<20	55	18.51	4.75	17.22	19.79	10.20	30.70		
LRA-EDV	>20	12	16.61	7.48	11.86	21.36	6.90	35.20	NS	−0.358 *P* = 0.003
	Total	67	18.17	5.33	16.87	19.47	6.90	35.20		

	<20	55	0.65	0.05	0.63	0.66	0.56	0.79		
RRA-RI	>20	12	0.73	0.07	0.68	0.77	00.65	0.86	0.0001	0.552 *P* = 0.0001
	Total	67	0.66	0.06	0.65	0.68	0.56	0.86		

	<20	55	0.66	0.06	0.64	0.67	0.53	0.77		
LRA-RI	>20	12	0.74	0.07	0.69	0.78	0.61	0.82	0.0001	0.477 *P* = 0.001
	Total	67	0.67	0.07	0.66	0.69	0.53	0.82		

	<20	55	1.13	0.17	1.08	1.17	0.87	1.57		
RRA-PI	>30	12	1.56	0.47	1.27	1.86	1.13	2.62	0.0001	0.604 *P* = 0.0001
	Total	67	1.21	0.30	1.13	1.28	0.87	2.62		

	<20	55	1.16	0.19	1.11	1.22	0.78	1.78		
LRA-PI	>30	12	1.56	0.35	1.33	1.78	1.01	2.22	0.0001	0.564 *P* = 0.0001
	Total	67	1.23	0.27	1.17	1.30	0.78	2.22		

**Table 6:** Significant correlations between liver and renal Doppler US parameters.

Surrogate markers of renal function	Parameters	Pearson correlation	*P*
	RRA-MnV	0.252	0.040
HA-PSV	RRA-PSV	0.347	0.004
	LRA-PSV	0.363	0.003

	RRA-MnV	0.250	0.041
HA-MnV	RRA-PSV	0.343	0.005
	LRA-PSV	0.355	0.003

	RRA-MnV	0.278	0.023
	LRA-MnV	0.266	0.030
HA-EDV	RRA-EDV	0.301	0.013
	RRA-EDV	0.287	0.019
	LRA-PSV	0.267	0.029

	RRA-RI	0.446	0.0001
	LRA-RI	0.528	0.0001
HA-RI	RRA-PI	0.387	0.001
	LRA-RI	0.451	0.001
	LRA-EDV	−0.250	0.041

	RRA-PSV	0.244	0.047
	RRA-RI	0.416	0.0001
HA-PI	LRA-RI	0.477	0.0001
	RRA-PI	0.344	0.004
	LRA-RI	0.394	0.001

A/P	RRA-PSV	0.284	0.020

**Table 7:** Significant correlations between surrogate markers of renal function and Childs and MELD scores, and renal Doppler US parameters.

Surrogate markers of renal function	Parameters	Pearson correlation	*P *
Serum urea	Child's score	0.353	0.012
	MELD score	0.435	0.002
	LRA-MnV	−0.294	0.036
	LRA-EDV	−0.313	0.025
	RRA-RI	0.297	0.035
	RRA-PI	0.296	0.035
	LRA-PI	0.280	0.046

Serum creatinine	RRA-MnV	−0.346	0.005
	LRA-MnV	−0.326	0.008
	RRA-EDV	−0.350	0.004
	LRA-EDV	−0.317	0.010

Serum sodium	Child's score	−0.423	0.001
	MELD score	−0.485	0.0001
